# National mortality trends associated with the death certificate–reported co-occurrence of malignant neoplasms and bacterial diseases in the United States, 1999–2023: a CDC WONDER retrospective analysis

**DOI:** 10.1097/MS9.0000000000005031

**Published:** 2026-05-08

**Authors:** Palwasha Asghar, Mohammad Sameed Safwan, Abdur Rehman, Sujata Lodh, Mahnoor Sheikh, Kamil Ahmad Kamil

**Affiliations:** a Department of Medicine, Khyber Medical College, Peshawar, Pakistan; b Department of Medicine, King Edward Medical University, Lahore, Pakistan; c Department of Medicine, Dr. KNS Memorial Institute of Medical Sciences, Barabanki, Uttar Pradesh, India; d Department of Medicine, Pak International Medical College, Peshawar, Pakistan; e Internal Medicine Department, Mirwais Regional Hospital, Kandahar, Afghanistan

**Keywords:** age-adjusted mortality rate, bacterial diseases, CDC WONDER, epidemiology, malignant neoplasms, mortality trends, multiple cause of death, United States

## Abstract

**Background and objectives:**

Growing evidence suggests that bacterial infectious diseases contribute substantially to morbidity and mortality among individuals with malignant neoplasms. However, data regarding mortality related to the co-occurrence of malignant neoplasms and other bacterial diseases is limited. This study analyzes the national mortality trends for the co-occurrence of malignant neoplasms and other bacterial diseases in the United States from 1999 to 2023.

**Methods:**

A retrospective study was conducted using Centers for Disease Control and Prevention Wide-ranging Online Data for Epidemiologic Research mortality data for individuals aged 15+ to calculate age-adjusted mortality rates (AAMRs) per 100 000 and annual percent change (APC) using joinpoint regression, stratified by year, sex, age, race/ethnicity, urbanization, and geographic regions.

**Results:**

From 1999 to 2023, 726 510 deaths were recorded involving malignant neoplasms co-occurring with bacterial diseases. The AAMR slightly declined from 8.14 in 1999 to 7.63 in 2012, followed by a rise to 10.02 in 2023 with an APC of 2.51 (95% confidence interval: 2.14–2.89, *P* < 0.05). Males had higher mortality rates than females, declining until 2012, then increasing, while female rates were stable before rising after 2013. West exhibited the greatest increase in AAMR. Non-Hispanic Black individuals had the highest absolute AAMRs throughout the study period. Nonmetropolitan areas showed greater increases in AAMRs compared to metropolitan areas. Older age groups, especially those 65 years and above, predominantly contributed to mortality.

**Conclusion:**

Despite improvements, mortality from bacterial diseases and malignant neoplasms has increased, particularly affecting older adults, men, racial minorities, and the western region, requiring targeted interventions to reduce disease burden in vulnerable communities.

## Introduction

Cancer is a disabling, challenging, and frightening disease that can affect any body part^[^1^]^. It is among the leading causes of mortality and morbidity worldwide and the second leading cause of death in the United States, with nearly two million new cases and a just over 600 000 deaths annually^[^2^]^. Beyond the human toll, cancer imposes immense socioeconomic costs through direct healthcare expenditures and indirect burden on caregivers. This persistent public health burden has fueled massive research efforts to unfold the preventable risk factors and modifiable contributors to cancer mortality^[^3^]^.

Among many contributors, infectious pathogens such as bacteria and viruses are identified as modifiable causes of cancer, accounting for 20% of human cancers^[^4,5^]^. Infectious organisms such as bacteria have emerged as key comorbidities influencing cancer outcomes as they interact with tumor biology, causing chronic inflammation, immune suppression, and have a direct role as genotoxic agents^[^6^]^. Among infectious agents, bacteria are thought to contribute to nearly 20% of all human cancers, highlighting the critical role of infection control in oncology^[^7^]^.

There are trillions of bacteria, viruses, and other organisms present in the human microbiome in a balanced proportion, and it has a central influence on not only homeostasis but also on disease predisposition^[^8,9^]^. Nonetheless, an imbalance between commensal and pathogenic bacteria through dysbiosis and/or bacterial infection can disrupt this equilibrium, leading to chronic inflammation and immune deregulation, which could potentially lead to tumor formation or progression^[^10^]^. In addition to causation, bacterial infection contributes to cancer morbidity by confounding treatment and inducing sepsis, leading to hospitalization in immunosuppressed patients^[^11,12^]^. Thus, understanding the national mortality trends of malignant neoplasms (C00–C97) co-occurring with other bacterial diseases (A30–A49) is vital to reflect the actual burden of Infection-associated neoplastic outcomes. However, few studies have examined this association comprehensively at the population level.

However, comprehensive, population-level data examining long-term national mortality trends specifically for the co-occurrence of malignant neoplasms and bacterial diseases (i.e. in this study, this refers to the concurrent listing of ICD-10 codes C00–C97 and A30–A49 on the death certificate) are limited. No study has utilized national death certificate data to analyze these trends over 25 years with detailed demographic and geographic stratification. Therefore, this study aims to answer the following research question: What are the national mortality trends for the co-occurrence of malignant neoplasms (C00–C97) and other bacterial diseases (A30–A49) in the United States from 1999 to 2023, and how do these trends vary by sex, age, race/ethnicity, urbanization, and geographic region? By analyzing data from the Centers for Disease Control and Prevention Wide-ranging Online Data for Epidemiologic Research (CDC WONDER) database, this study seeks to provide foundational insights into this evolving public health challenge, with implications for integrated prevention and treatment strategies. This study is reported in accordance with the STROCSS 2025 guidelines.

## Methodology

### Material and methods

This descriptive analysis drew on mortality data from the CDC WONDER platform. This study examines malignant neoplasia deaths among individuals aged 15–24 and above from 1999 to 2023, with subgroup analyses based on the presence of other bacterial diseases. Malignant neoplasia was identified using ICD-10 codes C00–C97, while other bacterial disease-related deaths were captured using ICD-10 codes A30–A49. Only decedents aged 15 and above at the time of death were included. Institutional review board approval was not required since the data was anonymous and publicly accessible. Mortality data were derived from the CDC WONDER database (CDC WONDER, Multiple Cause of Death 1999–2023)^[^13^]^ death certificates compiled across all 50 states and the District of Columbia. The study design and reporting followed the STROBE guidelines for observational research. A narrative review of the literature was conducted using PubMed to identify prior studies on mortality related to cancer and bacterial infections, which confirmed the absence of similar large-scale, long-term trend analyses.

### Data extraction

Data were categorized by year of death, sex (male/female), and 10-year age groups (15–24, 25–34, 35–44, 45–54, 55–64, 65–74, 75–84, ≥85 years). Geographic stratification was based on the U.S. Census regions (Northeast, Midwest, South, and West) and the 2013 National Center for Health Statistics Urban-Rural Classification Scheme. Metropolitan areas were urban areas, while nonmetropolitan areas were defined as rural areas. Race and ethnicity were categorized as Hispanic or Latino, non-Hispanic White, non-Hispanic Black or African American, non-Hispanic Asian or Pacific Islander, and non-Hispanic American Indian or Alaska Native. Records with missing demographic or regional identifiers were excluded from subgroup analyses to preserve data accuracy. Co-occurrence was defined as the presence of both malignant neoplasms (ICD-10 codes C00–C97) and other bacterial diseases (ICD-10 codes A30–A49) listed anywhere on the death certificate, irrespective of whether either condition was recorded as the underlying cause of death.

HIGHLIGHTS

- National mortality involving co-occurring malignant neoplasms and bacterial diseases was analyzed using Centers for Disease Control and Prevention Wide-ranging Online Data for Epidemiologic Research data from 1999 to 2023.
- After an initial decline until 2012, age-adjusted mortality rates increased significantly through 2023.
- Mortality rates were consistently higher among males, older adults, and non-Hispanic Black populations.
- The western United States and nonmetropolitan areas showed the steepest recent increases in mortality.
- Findings reflect population-level death certificate reporting patterns and are hypothesis-generating rather than causal.

### Statistical analysis

Age-adjusted mortality rates (AAMRs) per 100 000 population were calculated using the direct standardization method and standardized to the 2000 U.S. standard population. Temporal trends were analyzed using Joinpoint regression analysis (Joinpoint Regression Program, version 5.4.0; National Cancer Institute)^[^14^]^. A maximum of three joinpoints was allowed for each model, consistent with National Cancer Institute recommendations for long-term trend analyses. The optimal number and location of joinpoints were selected using permutation tests based on the Monte Carlo method. Annual Percent Change (APC) was estimated for each identified segment, and Average Annual Percent Change (AAPC) was calculated to summarize overall trends. Statistical significance was defined as a two-sided *P*-value <0.05 or when the 95% confidence interval (CI) did not include zero. As per CDC WONDER standards, data for subcategories with fewer than 10 deaths were suppressed to ensure stability and confidentiality, and these were excluded from the analysis.

## Results

### Overall mortality trends

Between 1999 and 2023, a total of 726 510 deaths were recorded across all age groups in the United States (central illustration). Although the AAMR decreased slightly from 1999 to 2012 (8.14 in 1999 and 7.63 in 2012) with an APC of −0.24 (95% CI: −0.56 to 0.08, *P* = 0.13), this change was not statistically significant. Afterward, between 2012 and 2023, the AAMR rose rapidly from 7.63 to 10.02 with an APC of 2.51 (95% CI: 2.14–2.89, *P* < 0.05). To further characterize these overall patterns, mortality trends were examined by sex, census region, race and ethnicity, urbanization level, and age group (Supplemental Digital Content Figure 1, available at: http://links.lww.com/MS9/B182; Supplemental Digital Content Table 1, available at: http://links.lww.com/MS9/B183).

### Mortality trends stratified by sex

Overall, AAMRs were consistently higher among males than females. In females, the AAMR remained stable from 1999 to 2013 (6.57 in 1999 and 6.33 in 2013) with an APC of −0.01 (95% CI: −0.33 to 0.33, *P* = 0.97). This slight change in APC was not statistically significant. Between 2013 and 2023, the AAMR increased from 6.33 to 8.49 with an APC of 3.01 (95% CI: 2.55–3.48, *P* < 0.05).

The AAMR among males decreased from 10.56 in 1999 to 9.65 in 2012 with an APC of −0.49 (95% CI: −0.83 to −0.15, *P* < 0.05). Afterward, the AAMR rose to 12.07 in 2023 with an APC of 2.21 (95% CI: 1.83–2.58, *P* < 0.05) (Supplemental Digital Content Figure 1, available at: http://links.lww.com/MS9/B182; Supplemental Digital Content Table 2, available at: http://links.lww.com/MS9/B183).

### Mortality trends stratified by census region

Regional analyses demonstrated heterogeneous mortality patterns. The Northeast experienced a statistically significant decline in AAMRs between 1999 and 2013, followed by a modest increase thereafter. The South and Midwest showed relatively stable or declining trends in the early period, with pronounced increases after 2011–2013. The West exhibited the steepest recent increase, particularly after 2019.

In the Northeast, the AAMR decreased slightly from 9.63 in 1999 to 8.06 in 2013 with an APC of −1.03 (95% CI: −1.50 to −0.56, *P* < 0.05). Afterward, the AAMR showed a gradual increase from 8.06 in 2013 to 9.46 in 2023 with an APC of 1.54 (95% CI: 0.81–2.26, *P* < 0.05).

The Midwest showed a slight decrease in AAMR from 7.19 in 1999 to 6.93 in 2013 with an APC of 0.01 (95% CI: −0.22 to 0.24, *P* = 0.95). This decrease in APC was not statistically significant. From there onward, the AAMR increased continuously from 6.93 in 2013 to 9.32 in 2023 with an APC of 2.77 (95% CI: 2.43–3.10, *P* < 0.05).

The AAMR in the South decreased from 8.33 in 1999 to 7.74 in 2011 with an APC of −0.41 (95% CI: −0.83 to 0.01, *P* = 0.05). This change approached but did not reach statistical significance. Afterward, the AAMR increased rapidly to 10.54 in 2023 with an APC of 2.85 (95% CI: 2.49–3.21, *P* < 0.05).

The West showed the greatest change in AAMR. Initially, the AAMR increased slightly from 7.36 in 1999 to 8.68 in 2019 with an APC of 1.14 (95% CI: 0.90–1.39, *P* < 0.05). The AAMR then increased to 10.21 in 2023 with an APC of 4.82 (95% CI: 2.61–7.08, *P* < 0.05). However, this post-2019 increase is based on a short time interval and should be interpreted cautiously, as it may reflect disruptions related to the COVID-19 pandemic (Supplemental Digital Content Figure 2, available at: http://links.lww.com/MS9/B182; Supplemental Digital Content Table 3, available at: http://links.lww.com/MS9/B183)

### Mortality trends stratified by race/ethnicity

The AAMRs across different racial groups, such as Hispanic or Latino, NH American Indian or Alaska Native, NH Asian or Pacific Islander, NH Black or African American, and NH White, were recorded. The AAMR among Hispanics or Latinos decreased a little from 7.95 in 1999 to 7.21 in 2012 with an APC of −0.25 (95% CI: −0.87 to 0.37, *P* = 0.41). This change was not statistically significant. The AAMR then increased to 8.92 in 2023 with an APC of 1.88 (95% CI: 1.32–2.44, *P* < 0.05).

Among NH American Indian and Alaska Native, the AAMR increased gradually from 6.33 in 1999 to 9.31 in 2023 with an APC of 2.50 (95% CI: 1.89–3.12, *P* < 0.05).

The AAMR among NH Asian or Pacific Islander decreased initially from 7.59 to 6.75 in 2019 with an APC of −0.20 (95% CI: −0.58 to 0.17, *P* = 0.27). This decrease was not statistically significant. Afterward, the AAMR increased to 7.85 in 2023 with an APC of 4.91 (95% CI: 1.73–8.18, *P* < 0.05); however, this estimate is based on a short post-2019 time segment and should be interpreted cautiously.

The AAMR among NH Black or African American decreased from 14.18 in 1999 to 11.51 in 2012 with an APC of −1.13 (95% CI: −1.56 to −0.71, *P* < 0.05).

Later, the AAMR increased to 13.12 in 2020 with an APC of 1.49 (95% CI: 0.52–2.47, *P* < 0.05). From 2020 to 2023, the AAMR increased to 15.00 with an APC of 5.13 (95% CI: 1.87–8.50, *P* < 0.05); however, this increase is based on a short time interval and should be interpreted cautiously as it might reflect disruption related to the COVID-19 pandemic.

The AAMR among NH White decreased a little from 7.49 in 1999 to 7.21 in 2012 with an APC of −0.11 (95% CI: −0.43 to 0.22, *P* = 0.50). This change was not statistically significant. The AAMR then increased to 9.6 in 2023 with an APC of 2.67 (95% CI: 2.31–3.00, *P* < 0.05).

Several recent increases, particularly those observed after 2020 in selected racial and ethnic groups and regions, are based on short time segments of 2–3 years. These short-term estimates may be influenced by reporting variability, healthcare disruptions, or mortality coding changes during the COVID-19 pandemic, and should therefore be interpreted with caution, as they may reflect disruptions related to the COVID-19 pandemic (Supplemental Digital Content Figure 3, available at: http://links.lww.com/MS9/B182; Supplemental Digital Content Table 4, available at: http://links.lww.com/ms9/b183).

### Mortality trends stratified by urbanization

AAMRs differed between metropolitan and nonmetropolitan areas, with generally higher and more variable rates observed in nonmetropolitan settings.

In metropolitan areas, the AAMR decreased from 8.28 in 1999 to 7.74 in 2013 with an APC of −0.29 (95% CI: −0.47 to −0.10, *P* < 0.05). Later, the AAMR increased to 8.47 in 2016 with an APC of 3.38 (95% CI: −0.12 to 7.0, *P* = 0.06), which was not statistically significant. The AAMR then further increased to 8.88 in 2020 with an APC of 0.90 (95% CI: −0.12 to 1.93, *P* = 0.08).

In nonmetropolitan areas, the AAMR decreased initially from 7.51 in 1999 to 6.93 in 2004 with an APC of −1.36 (95% CI: −2.86 to 0.16, *P* = 0.07). This decrease was not statistically significant. Afterward, the AAMR rose to 7.65 in 2013 with an APC of 1.18 (95% CI: 0.49–1.88, *P* < 0.05). A further rise in AAMR was recorded as 8.92 in 2016 with an APC of 5.42 (95% CI: −0.75 to 11.97, *P* = 0.08). This increase was not statistically significant. The AAMR then further increased to 9.46 in 2020 with an APC of 1.51 (95% CI: −0.19 to 3.23, *P* = 0.08). This change was also not statistically significant (Supplemental Digital Content, Figure 5 available at: http://links.lww.com/MS9/B182; Supplemental Digital Content, Table 5 available at: http://links.lww.com/MS9/B183).

### Mortality trends stratified by state

The AAMRs stratified by states showed that the District of Columbia had the highest average AAMR throughout the 25 years, followed by New Jersey, West Virginia, Mississippi, and Maryland (16.7, 11.03, 10.65, 10.51, and 10.45, respectively). The states with the lowest average AAMR included Alaska, Montana, Arizona, Idaho, and Maine (4.77, 5.44, 5.52, 5.64, and 5.65, respectively) (Supplemental Digital Content Figure 6, available at: http://links.lww.com/MS9/B182; Supplemental Digital Content Table 6, available at: http://links.lww.com/MS9/B183).

### Mortality trends stratified by age groups

Crude mortality rates were highest among adults aged 85 years and older, followed by the 75–84 and 65–74 age groups. While older age groups demonstrated statistically significant increases after 2013, mortality trends among younger adults were largely stable over the study period, with several age-specific APC and AAPC estimates demonstrating confidence intervals that included zero. These age groups showed a gradual increase in crude mortality rates throughout the study period. CMRs were much lower in the remaining age groups (the 55–64 age group, the 45–54 age group, the 35–44 age group, and the 25–34 age group) and the lowest among the 15–24 age group (Supplemental Digital Content Figure 4, available at: http://links.lww.com/MS9/B182; Supplemental Digital Content Table 7, available at: http://links.lww.com/MS9/B183, Supplemental Digital Content Figures 1, 2, available at: http://links.lww.com/MS9/B182).

## Discussion

This study offers a thorough examination of mortality trends linked to bacterial diseases and malignant neoplasms in the United States between 1999 and 2023. The findings describe changes in AAMRs across demographic, regional, and socioeconomic strata, based on national data from CDC WONDER. The steady increase observed after 2012 suggests emerging challenges at the nexus of oncology, infection control, and public health equity, even though early mortality declines are a reflection of advancements in early diagnosis, immunization programs, and antimicrobial therapy. The results emphasize the enduring impact of social determinants, comorbidities, and structural disparities in determining mortality outcomes, as well as the dual burden of bacterial disease and cancer in an aging population.

In line with the general downward trend in United States all-cause mortality during that time, there was a slight decrease in AAMRs from bacterial infections and malignant neoplasms between 1999 and 2012. This progress was made possible by advances in targeted therapy, including the introduction of immune checkpoint inhibitors and monoclonal antibodies, as well as improvements in screening and early detection^[^15^]^. It is concerning, though, that this trend reversed after 2012 and then sharply increased through 2023. The increase may be partially due to multimorbidity, environmental exposures, antibiotic resistance patterns, and the aging of the U.S. population, all of which increase the risk of infection in cancer patients.

Importantly, the sharp increases observed in the most recent years of the study period, particularly after 2020, should be interpreted within the broader context of the COVID-19 pandemic. The pandemic disrupted cancer screening, diagnostic pathways, and treatment continuity, while also altering healthcare utilization and mortality certification and coding practices across the United States. Several hypothesized mechanisms could explain the observed short-term mortality surges: (1) delayed cancer diagnoses and consultations leading to more advanced disease and increased susceptibility to infection; (2) shifting of healthcare resources away from routine cancer and infection management in immunosuppressed patients; and (3) potential changes in death certification where COVID-19 itself may be listed, potentially leaving underlying cancers or bacterial infections under-recorded. Therefore, the data for 2020–2023 should be viewed as potentially reflecting pandemic-related artifacts, and the sustainability of these trends will require verification with post-pandemic data^[^16^]^. These system-level disruptions may have disproportionately affected individuals with malignancy and concurrent bacterial infections, and may partially explain the short-term mortality increases observed across several demographic and regional subgroups.

A plateau in preventative and early detection programs among lower-income and rural populations, along with widening gaps in healthcare access, has been observed^[^17^]^. Because immunosuppressed oncology patients are more likely to experience infection-related complications and sepsis, the relationship between bacterial infections and cancer care is especially important. In order to prevent preventable deaths, these dynamics highlight the necessity of integrated infection surveillance in oncology settings.

Throughout the study period, male mortality was consistently higher than female mortality, reflecting both biological and behavioral factors. Due to past patterns of alcohol consumption, tobacco use, occupational exposures, and delayed screening participation, men are known to have higher incidence rates of cancer, especially for lung, liver, and gastrointestinal cancers^[^18^]^. Moreover, male immune responses are generally less effective in clearing bacteria but more pro-inflammatory, which may exacerbate infection-related complications^[^19^]^.

On the other hand, the increase in female mortality after 2013 might be linked to rising metabolic and lifestyle-related risks, including obesity, diabetes, and sedentary behavior, which increase the risk of both chronic infections and cancer^[^20^]^. This trend is further supported by the narrowing of the gender gap in some cancers, such as colorectal and lung cancer. Furthermore, there are still gendered barriers to healthcare use; women may experience unequal access to advanced therapies, under-representation in clinical trials, and delays in diagnosis. To reverse mortality trends, these findings collectively emphasize the significance of sex-stratified prevention strategies that address both lifestyle modification and equitable care delivery.

The Northeast had the lowest mortality over the 25 years, while the South and West had the highest AAMRs, according to an analysis of census regions. The high rates of smoking, obesity, and comorbidities, along with long-standing socioeconomic disparities and underfunded public health systems, are frequently blamed for the South’s disproportionate burden of chronic disease mortality, which is consistent with earlier CDC analyses^[^21^]^. Delays in diagnosis and less-than-ideal treatment continuity are caused by structural obstacles, such as a lack of tertiary-care facilities in rural Southern states, and inadequate oncology infrastructure may all have contributed to the observed increase in mortality in the Western region after 2019^[^22^]^. On the other hand, more robust public health systems, easier access to healthcare, and long-standing cancer screening initiatives could account for the Northeast’s relative stability. Mortality outcomes in the United States are still influenced by geographic differences in healthcare capacity, especially in early detection and palliative services.

These results indicate that long-standing systemic injustices are reflected in regional Differences in cancer- and infection-related mortality, which are not solely biologically driven. To address these, region-specific health policy strategies will be needed, such as community-based preventive programs, enhanced hospital capacity in high-burden states, and targeted resource allocation.

Throughout the analysis, racial and ethnic disparities were consistently identified as a determinant of mortality. Despite having the lowest relative increase over time, non-Hispanic Black people had the highest mortality rates. This paradox points to a combination of long-term structural disadvantage and partial gains from focused public health initiatives that have not closed the gap despite being successful in reducing disparities^[^23^]^. The high baseline mortality rate among Black Americans is consistent with a wealth of evidence that links socioeconomic deprivation to increased exposure to infection risk, delayed cancer diagnosis, unequal access to screening, and lower rates of curative treatment^[^24^]^.

After 2020, an increase in AAMR was observed among non-Hispanic Asian or Pacific Islander populations; however, this change is based on a short time segment and should be interpreted cautiously. This could be a result of better reporting procedures or changes in the burden of disease brought on by urban environmental factors, dietary modifications, and acculturation. Similarly, the combined effects of infectious comorbidities, inadequate insurance coverage, and linguistic and cultural barriers that impede access to healthcare may be the cause of rising mortality among Hispanic/Latino populations^[^25^]^.

The structural injustices ingrained in the American healthcare system are highlighted by the persistent racial disparities in mortality. Research indicates that minority groups are more likely to suffer avoidable infection-related complications and are less likely to receive cancer care that is guided by guidelines^[^26^]^. Stronger community-based infection control initiatives, fair access to precision medicine, and culturally sensitive outreach should all be incorporated into future interventions.

According to the current analysis, nonmetropolitan (rural) areas had higher AAMRs than their metropolitan counterparts. This urban-rural divide reflects larger trends in health inequality in the United States, where socioeconomic deprivation, a lack of specialists, and a higher cancer mortality rate are the main causes of poorer infection outcomes and higher cancer mortality among rural residents^[^27^]^. Rural hospitals frequently experience delayed diagnoses, fewer treatment options, and lower clinical trial participation due to a shortage of infectious disease and oncology specialists.

The amplitude of rural mortality is still higher even though the temporal trends in rural and urban areas are similar – first declining, then stabilizing, and then rising after 2012. These inequalities might be lessened by the growth of telemedicine, rural cancer networks, and federally funded mobile screening units. Outcomes may also be enhanced by fortifying infection control, oncology referral, and antibiotic stewardship programs in rural health facilities.

As expected, crude mortality rates were highest among older adults, particularly those aged 75 years and above. This is a reflection of the combined effects of polypharmacy, frailty, and biological aging. As the median age in the United States increases, there is an increasing public health concern about the convergence of bacterial disease risk and cancer risk in older populations. Age-related immune function decline, or immunosenescence, lowers resistance to cancer and infection^[^28^]^. At the same time, the risk of death is increased by age-related comorbidities like diabetes, heart disease, and chronic kidney disease.

Another factor that may contribute to the increasing mortality trends, particularly in the later years of the study, is the rising threat of antimicrobial resistance (AMR). Cancer patients, who are often immunocompromised and require frequent hospitalizations, are particularly vulnerable to infections caused by multidrug-resistant organisms. The increasing prevalence of resistant bacterial strains limits effective treatment options, potentially leading to higher rates of sepsis and mortality in this population. Future surveillance efforts must integrate data on AMR patterns to better understand their contribution to mortality in patients with malignant neoplasms.

These findings highlight the need for integrated geriatric care models that coordinate oncology, infectious disease management, and palliative support, as older adults already make up the majority of cancer deaths and hospitalizations related to infections. Future studies should concentrate on how multimorbidity clusters affect survival outcomes, as well as how cancer patients benefit from nutritional optimization and antibiotic prophylaxis.

### Public health implications

In U.S. public health, the intersection of bacterial diseases and malignant neoplasms represents a dual frontier that connects the epidemiology of infectious and chronic diseases. The increased death rate in recent years might be an indication that fragmented health systems are failing to address the multifaceted, overlapping vulnerabilities of cancer patients, especially those in rural or socioeconomically disadvantaged areas. The specific patterns observed in this study generate several targeted research questions. The sharp post-2019 increase in the Western U.S. warrants further investigation into region-specific factors such as healthcare infrastructure capacity, environmental exposures, or regional variations in AMR. Similarly, the persistently highest mortality rates among non-Hispanic Black populations call for in-depth qualitative and health services research to identify the specific barriers to timely diagnosis, equitable treatment, and infection prevention that perpetuate this disparity.

The pressing need for integrated surveillance systems that monitor infection and cancer outcomes in order to facilitate early intervention and resource allocation is one of the policy implications. To enhance real-time data sharing, programs like the CDC’s National Healthcare Safety Network and the National Cancer Institute’s SEER database could be connected. Increasing access to timely cancer screening, optimizing vaccination coverage (such as influenza and pneumococcal vaccines), and extending antimicrobial stewardship initiatives in oncology settings continue to be key components in lowering mortality.

Additionally, efforts to prevent infections and cancer must be centered on health equity. Preventing the cyclical amplification of disparities requires addressing upstream determinants, such as housing, poverty, environmental pollution, and healthcare access. Future research should build on these descriptive findings by employing more advanced analytical methods. For instance, machine learning and large language models could be leveraged to analyze unstructured data from death certificates or electronic health records to better understand the complex interplay of factors contributing to mortality, as has been explored in other areas of medical research^[^29^]^.

### Limitations

There are various restrictions on this analysis. First, CDC WONDER’s death certificate-based data rely on precise cause-of-death coding, which can change over time and between jurisdictions. Infection-related and cancer-related deaths can be mistakenly classified, particularly when both are contributing factors. Second, patient-level information like AMR profiles, cancer subtype, stage, and treatment plan is absent from the database. Third, mortality comparisons may be skewed by regional variations in healthcare access and reporting quality. Lastly, because this is a retrospective ecological study, it is impossible to determine causality, and trends may be influenced by unmeasured confounders.

The study’s nationwide reach, long duration, and stratified analyses, in spite of these drawbacks, offer important insights into changing mortality trends and identify important areas for intervention. Because this analysis is based on death certificate data and defines co-occurrence using multiple causes of death, the findings reflect population-level reporting patterns and do not establish a causal relationship between bacterial diseases and malignant neoplasms. Additionally, mortality patterns observed during 2020–2023 may reflect pandemic-related disruptions in healthcare access and death certification practices, which could influence short-term trend estimates. Additionally, the study is subject to the potential for misclassification of bacterial diseases on death certificates, particularly with the use of non-specific ICD-10 codes, and the database lacks information on specific bacterial pathogens, which precludes analysis of pathogen-specific mortality trends.

## Conclusion

In conclusion, this analysis shows that although improvements in infection control and oncology have helped some populations, since 2012, the national mortality rate from bacterial diseases and malignant neoplasms has increased, disproportionately impacting older adults, men, members of racial minorities, and people living in the Southern and Western United States. These results highlight the necessity of integrated, equity-focused strategies for preventing infectious diseases and cancer. Antimicrobial stewardship, universal access to early detection and treatment, and community-level health infrastructure should be the top priorities of future initiatives. Reversing these interconnected public health burdens will require ongoing surveillance and policy reform. As a descriptive study, these findings are hypothesis-generating and should prompt further analytical investigations into the drivers of these disparities to inform effective, targeted interventions.

## Data Availability

The dataset analyzed in this study is publicly available on the CDC WONDER online database (https://wonder.cdc.gov/). No special access permissions were required.

## Research registration unique identification number (UIN)

Not applicable. This retrospective analysis used publicly available, de-identified CDC WONDER data and was not prospectively registered.
